# An exploratory study of the potential learning benefits for medical students in collaborative drawing: creativity, reflection and ‘critical looking’

**DOI:** 10.1186/1472-6920-13-86

**Published:** 2013-06-17

**Authors:** Philippa Lyon, Patrick Letschka, Tom Ainsworth, Inam Haq

**Affiliations:** 1 Centre for Research and Development, Faculty of Arts, University of Brighton, 58-67 Grand Parade, Brighton BN2 0JY, UK; 2 Faculty of Arts, University of Brighton, 58-67 Grand Parade, Brighton BN2 0JY, UK; 3 Brighton and Sussex Medical School, Mayfield House, Falmer, Brighton BN1 9PH, UK

**Keywords:** Creativity, Drawing, Self-reflection, Interdisciplinarity, Collaboration

## Abstract

**Background:**

Building on a series of higher educational arts/medicine initiatives, an interdisciplinary drawing module themed on the human body was developed for both year 3 Craft students and year 3 Medicine degree students. This became the subject of a research project exploring how the collaborative approach to drawing adopted on this module impacted on the students’ learning. In this article, emphasis is given to issues thought to have most potential relevance to medical education.

**Methods:**

Using an ethnographic research design, the methods adopted were: direct observation of all aspects of the module sessions, audio and video recordings and photographs of the sessions, the incorporation of a semi-structured discussion at the end of each session, and anonymous student questionnaires.

**Results:**

A number of key themes emerged. The complex, phased and multi-sensory nature of the ‘critical looking’ skills developed through the drawing exercises was seen as of potential value in medical education, being proposed as analogous to processes involved in clinical examination and diagnosis. The experience of interdisciplinary collaborative drawing was significant to the students as a creative, participatory and responsive form of learning. The emphasis on the physical experience of drawing and the thematic use of the human body as drawing subject led to reflective discussions about bodily knowledge and understanding. There were indications that students had a meta-cognitive awareness of the learning shifts that had occurred and the sessions provoked constructive self-reflective explorations of pre-professional identity.

**Conclusions:**

This preliminary study suggests, through the themes identified, that there may be potential learning outcomes for medical students in this model of interdisciplinary collaborative drawing of the human body. Further research is needed to explore their applicability and value to medical education. There is a need to explore in more depth the beliefs, motivations and learning styles of medical students opting for the module, the significance and weighting of different learning and teaching elements in the module and the impact of the learning on medical students in the immediate post-module phase.

## Background

There have been, and still are, many connections in the principles and methods of art education and medical education. In both domains, drawing of the human form, whether through anatomical dissections, écorché figures or living human models, has been a means by which to acquire and convey knowledge [1-3]. It has been used to explore, understand and reveal the human body scientifically and aesthetically [4,5]. Within the humanities there is historical, theoretical and interpretive literature exploring the relationship between art/humanities and medicine, including a dedicated peer-reviewed journal (*Journal of Medical Humanities*, unpublished transcript) [6-8]. There are also a number of research projects, both quantitatively and qualitatively-designed, that have investigated the value of arts interventions in medical training to the development of the future doctor [9-16]. Many of these tend to focus on the possible benefits of a particular type of arts intervention on a specific clinical skill: the impact of arts ‘training’ on clinical observation skills, for example [17,18]. Within UK medical education, engagement with the arts has gained impetus in recent years, particularly through a range of arts-oriented Student Selected Components (SSCs). In parallel with this growth in arts/medicine teaching and research there has been, across a range of disciplines, increasing interest in the potential educational value of drawing [19,20].

The module that is the subject of this research study, ‘Human Body Form’ (HBF), is available as an option for 3rd year BA (Hons) Three Dimensional Design (Craft) students, referred to in the report by the shorthand ‘Craft’, and as an SSC for 3rd year Bachelor of Medicine Bachelor of Surgery (BMBS) students. Based in the University of Brighton, which unusually benefits from both a Medical School (run jointly by the Universities of Sussex and Brighton) and a Faculty of Arts, it emerged from a cluster of interdisciplinary arts/medicine learning, teaching and research developments and deploys the human body as a thematic focus for the interdisciplinary student group. This focus is flexibly realized through use of inanimate models of the human form, life models and clinical learning spaces designed for the scientific study of the body and anatomical specimens. At BSMS, the SSCs available are advertised to students by means of short descriptors. The descriptor for the HBF module stated that it would ‘focus on drawing as a method to investigate the form of the human body and how we interact with our surroundings through the senses.’ It also made clear that there were no pre-requisites and that the module would ‘involve BSMS and University of Brighton art and design students working together to develop a portfolio of drawings in response to guided workshop sessions and visits.’ This text identified to prospective students the focus on drawing as a method and a process, and indicated the collaborative approach that would be taken.

The human body theme was chosen due its relevance to both disciplines: whilst this may be self-evident with Medicine, it is also the case with Craft students, who make objects designed to appeal to, be interacted with or used by the human body. The premise of this module is that drawing not only engages and develops ‘skills’ of observation and dexterity but also other qualities key to the development of medical and craft professionals: creative and generative thinking; self-reflection; social awareness within the learning group; collaborative working and a sophisticated understanding of the process of looking to draw, referred to here as ‘critical looking’. The module focuses on drawing particularly as a method of investigating and reflecting on the understanding of the human body through the visual, and other, senses. Craft students taking the module do so voluntarily, to help them build up a collection of work for their final assessment portfolios; medical students are required to complete a reflective diary.

In 2012, the total student group of 11 registrations on HBF comprised 6 third year Craft students and 5 third year Medicine students. The gender distribution was Craft: 5 females and 1 male; Medicine: 3 females and 2 males. Details of students’ ages were not requested but it was known that two of the Medicine students were slightly older than the rest of the group, as they had discussed previous higher educational and/or professional work experience. Few of the Craft students had received formal drawing ‘training’, although several of them described engaging in drawing when planning or recording their processes of Craft making. Two of the Medicine students referred to being interested and involved in drawing out of personal interest prior to taking the module. Attendance overall was good. It should be noted that although Medical students have an 80% attendance requirement, Craft students have no attendance requirement for this module. The module spanned 8 weeks and used three different types of learning and teaching space: classrooms equipped with easels and drawing boards in one of the University of Brighton Faculty of Arts buildings at the Grand Parade site in Brighton; the Human Movement Laboratory, a facility used in teaching and research for allied health professional students and staff at the University of Brighton’s Darley Road site in Eastbourne; and the Brighton and Sussex Medical School’s Anatomy Laboratory on the University of Sussex campus at Falmer. Sessions were timetabled for Friday afternoons, 2–5.00. In the case of the sessions in the two laboratories, students were asked to convene earlier for travel. Sessions were organized around drawing exercises from 2 – 4.00 or 4.30, with a short break for putting away drawing equipment and making drinks. Approximately half an hour at the end of each session was used for gathering as a group for tea and coffee, whilst holding a discussion about experiences and learning from the session. Some sessions slightly overran due to students being keen to continue with a group discussion.

## Methods

The design of the research, which draws on an ethnographic research tradition, emerged from the researchers’ recognition of the complexity and plurality of human experience. More specifically, it arose from their recognition of the complexity of the attempt to understand and analyse the impact of drawing on the learning of an interdisciplinary group. The research team did not want to oversimplify the many issues, varied experiences and potential contradictions likely to emerge from such a study and anticipated that the impact of drawing on student learning would be challenging to identify and express. A preliminary survey of relevant literature had been carried out before the study commenced. In carrying out ethnographic research into the impact of collaborative drawing as an educational approach, it was considered important not only to consider the culture, learning and teaching practices and behaviour evident in the sessions but also to be alert to the power relationships inherent in such an educational research scenario, the different motivations, learning styles and creative histories of individual students and the hopes, interests and expectations of the teaching and research staff. The researchers wished to investigate the impact of collaborative drawing on a specific cross-disciplinary student group but also to explore whether theory might be developed from the research findings to inform further investigations. The researchers were keen to be open to issues and themes arising from the project that had not necessarily been anticipated but that were pertinent to the research question.

The research was designed around the 2012 HBF module, using four different methods to consider the impact of the collaborative drawing approach. These were: direct researcher observation of all sessions; recordings (audio, video and photographic) of sessions for transcription and analysis; semi-structured discussions; and anonymous questionnaires. The questionnaires were administered to all the students taking the module and were distinct from the reflective diaries Medicine students were required to complete for their assessment. The discussions, an adapted focus-group technique, were designed to support learning outcomes as well as provide opportunities for research data gathering, so that the latter was complementary rather than disruptive to the students’ learning. The methods were designed to help identify relevant and trustworthy information and provide checks against developing premature conclusions or single-researcher bias. Transcripts were open-coded by three individual researchers discretely, prior to a further joint phase of analysis that tested the identified themes to the point of saturation. Whilst the design of the content and the learning and teaching approach had been planned and prepared before the module commenced, the research team continually reflected throughout the module, not only on the impact of the drawing exercises but also on the impact of the research process on the student participants. This enabled the researchers to refine their focus group questions carefully and identify avenues for further exploration. No significant changes to the original study design were made during the period of the study. The team encouraged the students, as active participants in the research process, to be as vocal and involved as they wished about the aims and progress of the module and the research. The interim report of the research, illustrated with anonymised quotations, was circulated to the students for comment and feedback some months after the module had been completed. All but one student expressed their wish to be contacted in the future should any follow up to the research be developed.

Formal ethics approval for the study was granted by the Ethics Committee of the Faculty of Arts at the University of Brighton in November 2011.

## Results and discussion

### Critical looking

Many of the drawing exercises had been designed by the tutor to develop the students’ understanding of what is involved in the act of looking to draw and how multidimensional, concentrated and varied this is. A regular feature of warm-up drawing exercises in particular was the production of sketches in very short timeframes (10–30 seconds for some), drawing without looking at the page (‘blind’ drawing), drawing alternately with dominant and non-dominant hands, and rubbing out a sketch in order to draw again over its faint marks. These exercises shifted the emphasis from drawing as output - the notion that the main aim is the finished drawing – and onto drawing as a process of looking in depth and with full critical attention. Despite the advertised module descriptor, some of the medical students had initially viewed the module as an opportunity to ‘learn how to draw’, seemingly meaning either accurate objective drawing or an ‘art school’ approach to drawing aimed at developing a personal ‘style’. These notions were extensively unpacked in discussion. The HBF module approach was aimed in part at exposing the difference between drawing a notion of what ‘should’ be there, based on an assumption of what something looks like, and making a conscious effort to observe with critical and analytical awareness. This is an issue that has been identified and discussed in relation to object-based learning approaches in art and design [21]. As the students began to understand this meaning of looking to draw, they identified their own thoughts and ideas about how drawing might be relevant to them in their respective disciplines.

The process of looking to draw caused some medical students to reflect on the relevance to clinical procedures. In their HBF sessions they often had to repeat the act of looking at the same object many times, to think about what they were seeing and what was significant, constantly check and challenge the information gathered through previous acts of looking and then look again at an even finer level of detail for different elements, textures, lines, tonal values. One medical student made an analogy between this idea of deep, critical looking in order to draw, and the process of medical diagnosis. If a patient presented with a particular symptom, the student stated, you had to remain open to a wide range of factors and possible causes. There was a need to be aware, to question the obvious, to ‘see’ with the other senses: to listen, smell, palpate. Noting the value of the physical routines and repetitions involved in drawing, one medical student made another connection between drawing and medical education/practice:

We do have quite a lot of time where we have to go and practice things and it’s something that I’m definitely guilty of not doing enough, is actually practicing over and over again examining people. .. maybe we will be able to learn from drawing that it’s just a physical thing. And going over and over you change the way that you look. I’m not putting it very well. But when we learn an examination, so you have to look at eyes or ears, and then check all sorts of various things, and feel different bits, it is a very practical, very physical and very visual. And when we have to do palpation, and textures and things as well. And you can read it in a book but that’s not learning it (unpublished research transcripts of audio recordings taken from the Human Body Form module sessions. *HBF session 3, transcript 27.1.12*).

Here, as with other themes discussed below, the student’s comment is illustrative of a process of reflection and analysis through analogical relationships. Students did not tend to speculate overtly about the likely impact of the course on their future view of or role in medicine but displayed a range of deeper understandings and attitudes that had arisen from the collaborative drawing experience and the connections they had made with the practice of medicine. Thus the complex and multi-sensory nature of the ‘critical looking’ skills developed through the drawing exercises were, as in the example above, discussed in very animated terms as extremely important both in drawing and in clinical examination and diagnosis. This suggested that a stronger sense of the importance and complexity of gathering sensory information, processing and analyzing it and then developing this as a skill, had been gained.

### Interdisciplinary collaborative drawing

The interdisciplinary collaborative drawing approach provided a creative, participatory and responsive tool of learning. The collaborative (or ‘pair-drawing’) exercises varied in content and pairs consisted as far as possible of one student from each discipline [22]. In the early part of the module, specific pairings were established and sustained for two to three sessions. In discussion, the students indicated that it was more interesting if the pairs were regularly changed, as working with new partners created more fruitful and stimulating drawing experiences. The collaborative drawing process itself usually involved two students working on the same sheet of paper, at the same easel. Having been given the brief for the exercise by the tutor, they would be asked to start drawing. Whilst they were sometimes given rules governing their actions as a pair, such as allocating drawing to one individual and the power of editorial rubbing-out to another (with the latter officially designated as ‘in charge’) much of the time they were left to establish a method of working themselves. This meant finding a way to accommodate one another’s physical presence at the same easel, identifying a perspective/s to take, and deciding how and where to organize and coordinate their mark-making on the paper.

Students responded strongly to a number of the collaborative drawing exercises. Many of these exercises were described as particularly interesting and enjoyable, but some as difficult or disconcerting. Observations made of the students at work and analysis of their drawings and comments in discussion indicated a number of relevant factors. One of these was the clear tendency towards harmonization and conformity evident in the first session. Here, most of the collaborative drawings of the paper model skeleton, for example, had the appearance of a single individual’s drawings: students had accommodated their partner’s style of mark-making and adapted their own to such an extent that an almost entirely harmonious, singular ‘compromise’ approach had been wordlessly established. As the module progressed, students became far more confident and interested in exploring the productive dis-harmonies of drawing together, tending to retain their own typical weight and style of marks alongside or integrated with their partners. They found ways to produce drawings that incorporated difference or allowed some fragmentation. This was then a source of considerable interest in end-of-session discussions, with students focusing on the differences in approach to drawing as a creative and generative process and exploring different disciplinary perspectives.

Analysis of the collaborative drawing exercises indicated two main strands of interest. First, students experienced the collaborative drawing as a comparative and responsive participatory activity. Students were able to experience and consider the benefits of, for example, how their partners paced their work, how they stood to observe the drawing subject and how they approached scale and weight of line. They could see how each other selected aspects of the subject, often quite differently, for more detail or emphasis. Given the drawing paper was shared, there was a constant parallel process of instigating and responding to marks, developing new areas of the drawing and redirecting or finessing what was already there in response to the drawing partner. Second, a particular type of collaborative drawing exercise was engaged in to explore whether, and how, this could form a type of conversational communication [23]. Working in cross-disciplinary pairs, with each partner using a different colour, students made marks in response to each other, but without speech. Some students, as articulated in the end-of-session discussion, experienced this as meaningful communication in the style of a conversation. One, when asked about her drawing exercise with a partner said “I don’t know, there were times when I didn’t know what to say”, whilst another commented that “It was definitely like we were having a chat”. As the discussion of the exercise progressed another student volunteered: “I felt like it was question and answer, like a musical form”.

### Embodied experience

The module foregrounded both the human body as a subject for drawing and the physical character of drawing as a process. The nature of drawing as a deeply physical, sensory experience was highlighted on a number of occasions. The exhaustion experienced at the end of a session led to a discussion in which medical students expressed surprise at the extent to which drawing was ‘hard work’. Beneath this feeling of tiredness was the fact that students had been using many different aspects of their physical being in highly concentrated ways. The tutor’s drawing instructions involved detailed oral accounts of, for example, where the students’ gaze should be directed for a particular exercise, how a drawing implement should be held or how a particular medium could be applied to the paper in order to achieve different effects. This was often followed up by comments on the students’ interpretation and physical application of the instructions and sometimes a discussion of this, in which clarification was sought.

The descriptions of tasks continually emphasized not just physical technique but the gradual involvement or submergence of the body in the activity of drawing. The tutor frequently used both active verbs and sensory metaphorical language: ‘splash the tone on”, “the cartridge [paper] should bite the charcoal more”. Students’ absorption in their task was often conveyed in their stance at the easel: legs staked apart for stability around the easel legs, one arm relaxed or holding the edge of the drawing board, the other actively drawing, torso and head facing out towards the object being drawn: the whole posture at times looking like a kind of dance, or an embracing of the work itself. This apparent absorption in the task was not necessarily harmonious or relaxing, however. In one of the collaborative drawing exercises each partner had been given a distinct role, one as the drawer and the other as the eraser-editor of the emerging drawing:

I think it’s quite interesting how in a way it’s almost as though two components, the physical drawing and the cerebral component, the looking and the checking, and it’s almost like we are splitting these up. The ‘rubber’ person is doing the, the kind of comparison, the kind of cognitive part of it, the person with the stick is doing the physical part of it. And can you kind of use someone else’s brain, as it were, instead of your brain, you know? You are kind of like a beheaded arm (*HBF session 2, unpublished transcript 20.1.12*).

Here the reflective consideration of the physical, cognitive and emotional experience of the collaborative task highlights the complexity of the processes involved. The significance and potential applications of this reflective awareness are not clear from this study; for example, whether this might have any impact on the development of empathy about others’ embodied experiences. This is an issue for further research.

### Meta-cognitive awareness of learning

The students’ acquisition of drawing skills and techniques was shaped throughout not by tutor direction and individual practice alone, but by the interdisciplinary peer interactions and subsequent group debate with students and staff. There were indications that students were very aware of the learning shifts that had occurred and that these might be transferred to and deployed in other settings. As highlighted in the section above, students noted the significance of looking in the drawing sessions as a means of progressively developing observation skills and a continuously critical and creative frame of mind about what they were seeing. There was also a clear link with the way that repeated acts of looking and drawing, especially collaborative drawing, developed an appreciation of the different possible approaches to a problem or task: “And in medicine you have obviously other clinicians’ ways of doing things and you might say I like that, I like that way of doing that, and then in this group you might see somebody else’s and say I like the way they handled that, or . so maybe I can adopt that and adapt it” (*HBF session 3, unpublished transcript 27.1.12*). Some of the students identified, particularly during the haptic drawing session, that the process of collaborative drawing could help generate different, challenging ways of thinking about and expressing a subject. Again, whilst the students did not predict how this might impact on their role in the future, their observations were clearly and strongly related to their continual process of education and their deepening understanding of the many facets of being a medical practitioner.

There were critical points during the module when students made what they identified as significant shifts in the progress of their skills or understanding. In some cases these were common points of development for most of the students; in other cases they were points of progress for one disciplinary group more than the other, or even for a specific student. Several of the medical students had expressed, at the first session, an attachment to or belief in the importance of producing controlled, accurate, observational drawings, despite the module descriptor making no reference to such an aim: drawing experience was not a requirement, nor, indeed, relevant in any way. Quickly realizing that this was not the aim of the module and that the exercises would often be working against their preconceptions, they expressed their initial sense of discomfort and even shock: as one student put it, “Well, I don’t like making things up. When I do draw, which is very rarely, I tend to just obsessionally copy the thing, and it terrifies me, erm, departing from that. I feel like I’m being dishonest, or something. But no, it was quite fun…” (*HBF session 4, unpublished transcript 10.2.12*). Another student, also at the end of the first session, said “I just kind of have to say how annoyed I felt when we rubbed out our first drawing – the first time we had to rub out our drawing, I felt really annoyed then”. This comment attracted laughter in recognition of this shared moment of frustration, but it became clear that this ability to erase or relinquish drawings on a regular basis was a more significant challenge and shift in thinking for some students than others.

One session involved travelling to the University of Brighton’s Eastbourne campus to draw in the Human Movement Laboratory. The aim of this was to use an environment designed for scientific/clinical research as a contrast to the art school setting, so that drawing tasks could be set on the sense of human presence, or absence, within a harder, less comprehensible learning space. Whilst this created a different context (environment and atmosphere) for the drawing activity, the focus was still the theme of collaborative drawing investigations of ‘the body’. Students were informed about this shift in locations in advance and were very interested in the new and different perspective and experience this could offer. As in all sessions throughout the 8 week course, students were encouraged to interact with staff, comment on their experience and reflect in some depth afterwards. In the case of the Human Movement Laboratory session, whilst the students initially appeared to relish the prospect of a journey together and the use of an unfamiliar venue for the drawing, they were not only disconcerted by the length of the trip and the different atmosphere of the campus but confused by the nature of the laboratory itself. It was in this space that students were asked to conduct the conversational drawing exercises in pairs, and then to create ‘body absent’ compositions that might suggest humanity, without it being physically present. In the discussion about how this session had been experienced, the students were predominantly negative. The observation from one student was fairly typical: “I didn’t feel comfortable drawing because it is a bit of a confusing place. I didn’t know what the room was and I guess I was a bit more timid in how I drew. So I didn’t maybe draw as well because I felt this is a really strange place and I don’t really know what I am drawing. I didn’t enjoy it as much” (*HBF session 4, unpublished transcript 10.2.12*). Deeper into the discussion, some of the students said that they felt the session had been useful, but they had not enjoyed it. The research team were, however, struck by the contrast between the students’ lack of overall enjoyment of the session and how promptly, cooperatively and creatively they had engaged in the tasks and how distinctive and interesting their drawings had been. It provided one of the strongest examples of how challenging and discomforting moments in the learning and teaching prompted thoughtful, different approaches, a complex issue that has been noted and explored elsewhere [24-26] and one that merits careful further investigation in relation to collaborative drawing. Furthermore, the discomforting experience of this session was recalled by the students as particularly memorable during the final critical review session. This suggested that although experienced as uncomfortable, this particular session was significant in helping students to recognize the emotional complexities experienced by patients when consulting health professionals in clinical environments and the effect that this can have on the patients’ perception of the overall experience. The reactions observed on the HBF course are not taken to have implied an inevitable causal relation between challenge or discomfort and learning, however, and clearly many other factors were involved.

### Identity

The sessions provoked a range of constructive self-reflective explorations of pre-professional identity. Through observation and discussion during the module, it became clear that the significance of students’ developing disciplinary and professional (or pre-professional) identities in the sessions was considerable. As discussed elsewhere, ‘Who we are, and who we are seen to be, underlies much of what we do in medical education’ [27]. This was presented in a number of different ways, ranging from physical and sartorial display, articulation of identity issues in the formal feedback mechanisms and end-of-session discussions and conversational allusions in the form of humour and banter. Interestingly, whilst the research team members had discussed prior the start of the project how they might handle a situation where the respective groups of students might have conflicting views of each other’s status, or enact a sense of superiority or inferiority triggered by the cross-disciplinary setting, this was not evident beyond the establishing of the group in the first session (in which some Medicine students indicated that they felt at a disadvantage in drawing skill by comparison with the Craft students). The Craft students, whilst perhaps initially at some advantage due to being on their own art school ‘territory’, quickly displayed a respect and interest in the act of drawing collaboratively with the Medicine students, with the latter often quick to debunk or satirize any associations of privilege and power with Medicine. Issues of power and status were referred to, but either within end of session discussions, or through processes of social interaction. This was evident both during the drawing part of the sessions and during the sociable and informal pause as tea and coffee was made prior to the group discussion. In terms of physical and sartorial display, one of the medical students attended the first session in an item of clothing printed with a full-size skeleton design, which became a talking point and was worn and referred to in later sessions. Another of the medical students talked of keeping the dark charcoal marks he had on his face from the drawing activity so that he could indulge in giving an impression of being an artist or art student. To some extent, identity-oriented behavioural and conversational strategies are to be expected from many newly formed learning groups. The types of display observed were quite distinctive in character, however, and reflective of the intersection of the two disciplines concerned: often playful but occasionally and abruptly becoming earnest; attracting explicit and often physical attention to the respective subject affiliations; and provoking challenging but thoughtful discussion about disciplinary identity issues. In the more formal taught part of the sessions, students displayed a very sophisticated and reflective interest in the issue of what it meant to be a medical student or craft student both in general, and personally. Also identified were different perceptions of the value and use of drawing. Medical students generally thought of drawing as a learning tool that was particularly relevant to anatomy, dependent on accuracy and detail. Crafts students had a tendency to use drawing for a wider range of functions: expressively, through abstract mark making; for accuracy, when recording observations or working out details of a making project; or open-endedly, as a means to explore and identify opportunities for further investigations.

Reflection on identity issues came across particularly strongly during session discussions. In the initial two sessions where the group established itself and became acclimatized to the learning and teaching approach, students engaged in a number of teasing exchanges with each other. In a protracted piece of conversation in session two, in between drawing exercises and the more structured group discussion, students mock-argued about the relative qualities and benefits of being in their respective disciplines: ‘Doctors against artists’. This was humorous, even darkly humorous, in nature: “Who would win?”, “We’ve got scalpels”, “We’ve got saws as well” (*HBF session 2, unpublished transcript 20.1.12*). It was clear in this exchange that the students’ mutual interest in and respect for each other’s discipline had already been firmly established and that they were enjoying this process of verbal play fighting. There was no evidence of malice, rather an amusement in the contrasts and similarities between the two disciplines and an establishment of boundaries for social interaction. Humour was often used in this way to express and assert identity and also, occasionally, to explore and justify behavior, knowledge and their approach to learning. Whilst at times students made it clear they did not welcome being stereotyped according to their discipline, or were keen to qualify the impressions that those from other disciplines had of them, the overall pattern was towards students finding a way that suited them of ‘wearing’ their disciplinary identities overtly. The research team was extremely conscious throughout of their potential influence in emphasizing or reinforcing disciplinary distinctions and differences. Part of the research team’s ongoing reflective consideration of the drawing sessions concerned the need to avoid giving any sense of forcing students to identify themselves by their discipline. Whilst regular references were made to ‘Craft’ and ‘Medicine’ students during sessions, given this was integral, for example, to the approach to pairing students for collaborative drawing activity, great care was taken to avoid any sense of enforcing specific subject-defined identities. Students were invited to explore and challenge the disciplinary and professional identities they associated with their respective courses of study. They grasped this opportunity, with several of them discussing at some length the insights they had gained into their discipline, not just from stepping back from their usual course work but from actively engaging in collaborative, interdisciplinary and discursive learning through drawing.

The explorations of identity were only partly parodic in outward form. There were many instances where students invoked aspects of their disciplinary knowledge in more serious ways. It was not always clear whether these instances involved intentional sharing with or display to the group, or a natural means of understanding and discussing their experiences. As the group drew the paper model of a skeleton in the first session, for example, one of the medical students observed that “there’s a little bone coming away from its neck. It’s been bothering me” (*HBF session 4, unpublished transcript 10.2.12*). An exchange with other students, the tutor and the researchers ensued, resulting in the correct placement of the stray paper bone and a discussion of the sex of the skeleton. In many such instances, students showed how steeped they were in their disciplinary studies and how it affected their behaviour; it was clear that their studies shaped who they were to a large extent. Their knowledge at times functioned as a kind of currency to share with and benefit others in the group, and they sometimes became aware or were reminded of tacit skills and knowledge they possessed in a way that enabled them to appreciate it anew.

Yet at other times, whilst drawing, medical students chose not to, or were not able to use their specialist knowledge. The Faculty of Arts staff and students had been curious from an early stage about whether the medical students’ specialist knowledge would be a constant presence, informing their actions and interpretations throughout the module. The medical students made a number of points at different times in response to this expectation. They sometimes viewed the drawing sessions as a valuable and enjoyable contrast to their usual medical education, and whilst their specialist medical knowledge and language were invoked at times, there were many other occasions where drawing simply took precedence. One medical student commented that it was simply not possible for him to refer back to his medical knowledge whilst involved in the drawing exercises: this wasn’t just a personal preference but a sense of being ‘at capacity’.

Identity became a more emotive issue at points in the module. This, for some students, was a process of exposing and exploring their personal relationship to the general, societal perception of an artist or medic. The question of identity raised issues of limitation, fulfillment and freedom: did the prospect of becoming a craftsperson or medic feel a full, rich and desirable prospect, or did it feel like a process of becoming pigeonholed, even misunderstood? Was there a point at which the students had made a fully conscious, self-willed decision to pursue their respective disciplines/professions, or did they feel that this had emerged more passively? The researchers had not anticipated that these issues would emerge so strongly. It became a fruitful area of debate, returned to on a number of occasions by the students. In session one, for example, as the tutor and researchers were not yet fully aware of some of the sensitivities about identity, one student mused: “I don’t really find that I really .. medical student, I don’t just feel like ‘a medical student’, I am just a person” (*HBF session 4, unpublished transcript 10.2.12*). This comment is particularly interesting in view of medical education debates in the UK concerning the need for a holistic perspectives and understanding [28] and the potential benefits of some arts-based methods and approaches within selected areas of medical education [29].

The students made many insightful observations about the connections and resonances between Medicine and Craft. Both disciplines, as one student put it, were equally fascinated by ‘the mundane and the bizarre’. Their developing professional identities had to accommodate the fact that they were likely to be engaged with ordinary, repetitive tasks and the mundane details of everyday life, that they needed to be interested in and value, the quotidian. Yet equally, a fascination with the unusual, whether physiological, behavioural or artistic, was felt to connect the two disciplines.

## Conclusion

This research was carried out as a first, formal attempt at the University of Brighton to take a particular range of collaborative Arts/Medicine educational initiatives onto a different footing. In presenting this, the researchers are mindful of the larger debates around the methodologies of such research and need to consider impact on attitudes and behaviour in the longer term [30]. One of the aims of the research team is to take the findings of this project into future research, to consider whether and how collaborative educational drawing approach might impact on students in their future professional practice. However, as a result of the project, a number of productive key themes were identified. The students involved in the collaborative drawing underwent distinct shifts in their learning, not only in terms of challenging their assumptions about drawing and critiquing their notions of ‘good drawing’ but also in realising that through drawing, they were brought into closer relationship and understanding with the human body. Furthermore, there were indications that some of the aspects of the module that students experienced as unsettling or not directly enjoyable nevertheless led to new insights and learning outcomes; an observation that needs to be investigated in more detail, and discussed in the context of other literature in the field. The students extended their ability to look in great depth and use different senses in order to draw and they developed analogous awareness of how this ‘critical looking’ could be applied in other situations. The comparative and participatory quality of collaborative drawing enabled students to extend their learning through working with and alongside others and to begin to investigate the issues around drawing as communication. Students developed a reflective awareness of the physicality of drawing and the experience of tapping strongly into senses other than the visual. The reflective, discursive mode that students habitually adopted on the course led to a high level of awareness of their own learning and important issues of disciplinary and pre-professional identity were exposed and developed.

In combing out these themes, the researchers have set out on an exploratory path, where the attempt to identify the learning outcomes of drawing in interdisciplinary higher education settings has propelled them into a range of further problems and questions that need to inform future research. Whilst ethnographic research carried out in specific, naturalistic educational settings is not generalisable in the sense that positivist or empiricist research might claim, the findings are felt to be sufficiently interesting and potentially beneficial for a further study to be run. The researchers will be carrying out a second formal research project into the collaborative drawing approach at Brighton to gauge whether there is a recurrence of any or all of the themes. It is suggested that the approach outlined here, where a collaborative drawing module themed on the human body is designed to be taken as SSC as well as an optional module for craft students, could also be adapted for use, development and possible research in other medical schools, taking into account local learning and teaching approaches and practices as necessary. It is also suggested that the approach outlined in this paper could be further adapted and tested for use with nursing and allied health professionals, to explore whether the issues and potential benefits articulated here are also relevant to these groups.

## Competing interests

The authors declare that they have no competing interests.

## Authors’ contributions

PL (Patrick Letschka) and TA developed, implemented and taught the HBF module including development of a range of drawing exercises. This built on initiatives and previous design/medicine interdisciplinary learning and teaching research carried out by IH and TA. IH, through BSMS, provided support for HBF to be offered to medical students as an SSC. PML (Philippa Lyon) developed the research design with TA and PL. TA wrote the ethics application. PML carried out the literature review and PL, TA and PML jointly oversaw the data collection. PML developed and oversaw the qualitative analysis process; PL, TA and PML carried out analysis of the data independently, then discussed and agreed themes jointly. PML had primary responsibility for writing the final article. IH offered comments and insight from a Medical Education perspective. All authors reviewed and approved the final article.

## Authors’ information

Philippa Lyon is a Research Fellow in the Faculty of Arts, University of Brighton.

Patrick Letschka is a Senior Lecturer in the School of Art, Design and Media, Faculty of Arts, University of Brighton.

Tom Ainsworth is a PhD student and lecturer in the Faculty of Arts, University of Brighton.

Inam Haq is Director of Undergraduate Studies and Head of the Medical Education Unit at the Brighton and Sussex Medical School.

## Pre-publication history

The pre-publication history for this paper can be accessed here:

http://www.biomedcentral.com/1472-6920/13/86/prepub
